# Ethylene regulates post-germination seedling growth in wheat through spatial and temporal modulation of ABA/GA balance

**DOI:** 10.1093/jxb/erz566

**Published:** 2019-12-24

**Authors:** Menghan Sun, Pham Anh Tuan, Marta S Izydorczyk, Belay T Ayele

**Affiliations:** 1 Department of Plant Science, 222 Agriculture Building, University of Manitoba, Winnipeg, Manitoba, Canada; 2 Grain Research Laboratory, Canadian Grain Commission, Winnipeg, Manitoba, Canada; 3 Royal Holloway, University of London, UK

**Keywords:** Coleoptile, embryo axis, gene expression, germination, plant hormones, root, seedling, starch degradation

## Abstract

This study aimed to gain insights into the molecular mechanisms underlying the role of ethylene in regulating germination and seedling growth in wheat by combining pharmacological, molecular, and metabolomics approaches. Our study showed that ethylene does not affect radicle protrusion but controls post-germination endospermic starch degradation through transcriptional regulation of specific *α-amylase* and *α-glucosidase* genes, and this effect is mediated by alteration of endospermic bioactive gibberellin (GA) levels, and GA sensitivity via expression of the GA signaling gene, *TaGAMYB*. Our data implicated ethylene as a positive regulator of embryo axis and coleoptile growth through transcriptional regulation of specific *TaEXPA* genes. These effects were associated with modulation of GA levels and sensitivity, through expression of GA metabolism (*TaGA20ox1*, *TaGA3ox2*, and *TaGA2ox6*) and signaling (*TaGAMYB*) genes, respectively, and/or the abscisic acid (ABA) level and sensitivity, via expression of specific ABA metabolism (*TaNCED2* or *TaCYP707A1*) and signaling (*TaABI3*) genes, respectively. Ethylene appeared to regulate the expression of *TaEXPA3* and thereby root growth through its control of coleoptile ABA metabolism, and root ABA signaling via expression of *TaABI3* and *TaABI5*. These results show that spatiotemporal modulation of ABA/GA balance mediates the role of ethylene in regulating post-germination storage starch degradation and seedling growth in wheat.

## Teaser

Ethylene controls post-germination starch degradation and seedling growth, but not radicle protrusion, in wheat through spatiotemporal alteration of the balance between abscisic acid and gibberellin.

## Introduction

Seed germination and seedling growth are complex physiological processes regulated by several plant hormones. Abscisic acid (ABA) and gibberellin (GA) are the major players in this regard; ABA inhibits seed germination and seedling growth while GA promotes these early developmental processes (Finkelstein *et al.*, 2002; Sun, 2008; Shu *et al.*, 2018; Tuan *et al.*, 2018). These effects of ABA and GA are mediated partly by their levels, which are determined by the balance between their biosynthesis and catabolism. The level of ABA in plant tissues is regulated mainly by the actions of 9-*cis*-epoxycarotenoid dioxygenase (NCED) and ABA 8'-hydroxylase (ABA8′OH; encoded by *CYP707A* genes) that catalyze ABA biosynthesis and catabolism, respectively (Nambara *et al.*, 2010), while the level of GA is controlled mainly by the actions of GA 20-oxidase (GA20ox) and GA 3-oxidase (GA3ox) (GA biosynthesis), and GA 2-oxidase (GA2ox) (GA catabolism) enzymes (Yamaguchi, 2008). The effects of ABA and GA are also mediated by tissue sensitivity to these hormones, which is regulated by their respective signaling pathways. ABA signaling involves several components, including the downstream transcriptional regulators ABI3 and ABI5 (Nambara *et al.*, 2010), while the growth-repressing DELLA protein and downstream transcription factor GAMYB represent important components of GA signaling (Sun and Gubler, 2004). Genetic and mutational analyses of the ABA and GA metabolism and signaling genes have shown their importance in regulating seed germination and seedling growth (Finkelstein *et al.*, 2002; Sun, 2008; Nambara *et al.*, 2010; Matilla *et al.*, 2015).

Other phytohormones such as ethylene (ET) also regulate seed germination and seedling growth. ET promotes germination in dicot species, while inhibition of ET synthesis is associated with repression of germination (Arc *et al.*, 2013; Corbineau *et al.*, 2014). The synthesis of ET in non-dormant seeds of both dicots and cereals starts with imbibition and peaks during radicle emergence (Lalonde and Saini, 1992; Petruzzelli *et al.*, 1994; Kucera *et al.*, 2005), and this is associated with the expression patterns of ET biosynthetic genes (Linkies and Leubner-Metzger, 2012). In contrast, neither inhibition of ET synthesis nor treatment with an ET precursor or ET-releasing compound affects the germination of non-dormant cereal seeds (Locke *et al.*, 2000; Gianinetti *et al.*, 2007). ET has also been implicated in regulating seedling growth; it inhibits root elongation in dicot seedlings (Ruzicka *et al.*, 2007; Dong *et al.*, 2011), and its effect on hypocotyl elongation depends on light as it exhibits inhibitory and stimulatory effects in the dark and light, respectively (Smalle *et al.*, 1997). In cereal seedlings, ET promotes coleoptile elongation irrespective of light condition but inhibits root growth (Yin *et al.*, 2017), while inhibition of ET synthesis represses the elongation of both tissues (Gianinetti *et al.*, 2007).

The role of ET in regulating seed germination and seedling growth is mediated at least partly by its antagonism of ABA effects. For example, seed germination and seedling root growth in Arabidopsis mutants with enhanced ET synthesis and sensitivity such as *ethylene overproducer1* (*eto1*) and *eto3*, and *constitutive triple response1* (*ctr1*) are less sensitive to ABA (Beaudoin *et al.*, 2000; Ghassemian *et al.*, 2000). On the other hand, ET deficiency due to mutation in the ET biosynthetic gene *ACS7* promotes seed sensitivity to ABA and thereby inhibition of seed germination and seedling growth (Dong *et al.*, 2011). Furthermore, mutants with reduced ET sensitivity such as *ethylene resistant1* (*etr1*) and *ethylene insensitive2* (*ein2*) exhibit increased ABA content and sensitivity, leading to inhibition of germination (Arc *et al.*, 2013). Seedling root growth in these mutants, however, is less sensitive to ABA (Ghassemian *et al.*, 2000), suggesting the requirement for a functional ET signaling for inhibition of root growth by ABA. In cereal seedlings, ET-induced inhibition of root growth is reported to be mediated by an enhanced ABA level, while inhibition of coleoptile elongation by ABA is mediated by repression of the ET response (Ku *et al.*, 1970; Ma *et al.*, 2014).

Interplay between ET and GA has also been implicated in the regulation of germination and seedling growth. For example, inhibition of ET synthesis and signaling in imbibing dicot seeds alters the expression patterns of GA metabolism genes and thereby inhibits germination (Iglesias-Fernandez and Matilla, 2010), and such effects of a reduced ET level can be reversed by GA (Calvo *et al.*, 2004*b*). Conversely, GA promotes ET synthesis and germination via up-regulating ET biosynthetic genes and enzymes, and ET can reverse the effects of decreased GA levels (Calvo *et al.*, 2004*a*). Previous studies in Arabidopsis seedlings showed the requirement of GA for ET-mediated stimulation of apical hook formation that occurs through enhanced cell division and elongation (Vriezen *et al.*, 2004) while inhibition of root growth by ET is DELLA dependent (Achard *et al.*, 2003). Furthermore, the role of ET in promoting seedling elongation in rice appeared to be mediated through enhanced GA response (Furukawa *et al.*, 1997). Consistently, GA promotes hypocotyl or coleoptile elongation (Cowling and Harberd, 1999; Konishi *et al.*, 2005), and such an effect of GA is mediated by up-regulation of genes encoding expansins such as α-expansin (EXPA), proteins that act as major regulators of cell enlargement (Sampedro and Cosgrove, 2005). Despite these reports, the molecular links underlying the role of ET in regulating ABA/GA balance, and thereby germination and seedling growth, in cereals are poorly understood.

Post-germination seedling growth is also regulated by mobilization of seed storage reserves that serve as a primary source of energy, and mutations that compromise mobilization of seed storage reserves inhibit seedling establishment (Penfield *et al.*, 2005). In seeds of cereals, endospermic starch serves as a major storage reserve, and its mobilization involves the actions of α-amylase (AMY), which directly acts on starch granules and produces branched and linear glucans that are further hydrolyzed into glucose by the combined actions of debranching enzyme limit-dextrinase, β-amylase (BAM), and α-glucosidase (AGL). Activation of these enzymes during imbibition is associated with reduction in the endospermic starch level and increases in the levels of soluble sugars such as glucose, fructose, and maltose (Palmiano and Juliano, 1972). It is well established that starch degradation during imbibition of cereal seeds is regulated antagonistically by ABA and GA; for example, the expression of starch-degrading genes such as *AMY* is enhanced by GA but repressed by ABA (Gubler *et al.*, 1995; Gómez-Cadenas *et al*., 1999, 2001). Although earlier reports implicated ET in the control of amylase synthesis and release in the aleurone tissues of cereal seeds (Eastwell and Spencer, 1982; Varty *et al.*, 1983), its role in regulating the degradation of endospermic starch and the underlying molecular bases remain to be elucidated.

Overall, previous studies that provided insights into the molecular mechanisms of ET interplay with ABA and GA during germination and seedling growth were focused mainly on dicot species; therefore, much less is known about this phenomenon in cereals, particularly in the polyploid wheat as the genomic/reverse genetic resources that are crucial in elucidating such mechanism are still very scarce. To this end, this study investigated the molecular bases underlying the role of ET in regulating spatiotemporal modulation of ABA/GA balance, and thereby germination, storage starch degradation, and seedling growth in wheat.

## Materials and methods

### Plant materials, germination, and seedling growth assays

Wheat genotype RL4452, which produces non-dormant seeds at maturity, was used for this study (Yamasaki *et al.*, 2017). Mature seeds were harvested from plants grown under conditions described previously (Izydorczyk *et al.*, 2018). Seed surface sterilization and germination assays were undertaken as described previously (Gao *et al.*, 2013). To examine the effect of ET on germination and seedling growth, seeds were imbibed with 1 mM aminoethoxyvinylglycine (AVG), an ET biosynthesis inhibitor (Cayman Chemicals, Ann Arbor, MI, USA), 10 μM ethephon (a compound that releases ET), or a combination of 1 mM AVG and 10 μM ethephon. Three AVG concentrations (100 μM, 500 μM, and 1 mM) were tested initially, and 1 mM AVG was chosen as it was most effective in inhibiting seedling growth without any toxic effect; 10 μM ethephon was chosen as higher concentrations caused inhibition of root growth. To investigate if the effect of AVG on germination and seedling growth is mediated by changes in the levels of GA and ABA, both control and AVG-treated seeds were imbibed (25 seeds per plate per replicate, three replicates) with 50 µM GA_3_ (Sigma-Aldrich, St. Louis, MO, USA) or 50 μM fluridone (ABA biosynthesis inhibitor; Sigma-Aldrich) for 7 d.

Scoring of seed germination, which was defined by coleorhiza emergence through the seed coat, and measurement of the length of different seedling parts (embryo axis, coleoptile, primary root, and seminal root) were performed over a period of 7 d; the coleoptiles did not form any leaves during the study period. For gene expression and hormone (ABA, GA, and ET) level analyses, seeds were harvested (25 seeds per plate per replicate, three replicates) at 0, 1, 3, 5, and 7 days after imbibition (DAI) and separated into ‘endosperm’ (including aleurone and pericarp) and ‘embryo axis’ (including scutellum) at 0, 1, and 3 DAI, and into endosperm, coleoptile, and ‘root’ (including primary and seminal roots) at 5 and 7 DAI. The activity of starch-degrading enzymes, and the levels of starch and soluble sugars were also determined in the endosperm samples. To determine the origin of endospermic bioactive GA and its potential effect on the endospermic ABA level during imbibition, embryo axes were excised before the start of imbibition and the endospermic GA and ABA levels were determined at 5 and 7 DAI, when high GA accumulation was evident in the endosperm of control seeds (see below). All tissues were immediately frozen in liquid nitrogen after harvest and then stored at –80 °C until further use.

### Ethylene measurement

The ET level was analyzed in the different tissues as described previously (Geisler-Lee *et al.*, 2010) with minor modifications. Briefly, endosperm (1, 3, 5, and 7 DAI), embryo axis (1 and 3 DAI), and coleoptile and root (5 and 7 DAI) tissues were excised from imbibing seeds/seedlings and placed between two moist filter papers for 1 h to minimize the potential effects of wounding. Each sample was then transferred to a 10 ml glass tube containing a sterile 1 cm^2^ Whatman #1 filter paper moistened with 100 μl of sterile water to maintain hydration of the tissues, and the tubes were tightly closed with a rubber septum. After 3 h incubation, 3 ml of head space was removed from the tube with a gas-tight syringe and then injected onto a Bruker 450-GC gas chromatograph.

### Identification of wheat starch-degrading and *GAMYB* genes and their specific primers

Available sequences of barley genes encoding starch-degrading enzymes (AMY, BAM, and AGL) and the GA signaling gene *TaGAMYB* were used to search the corresponding wheat homologs in the National Center for Biotechnology Information (NCBI) wheat UniGene database using the basic local alignment search tool (BLAST). The resulting UniGene sequences were then BLAST searched against the wheat genome sequence data in Ensembl Plants (http://plants.ensembl.org/Triticum_aestivum/) to identify the respective full-length sequences of the target genes and proteins. The resulting sequences of wheat starch-degrading and *TaGAMYB* genes were used as queries to blast search the respective orthologs in the NCBI database. Gene/homolog name assignment was based on their orthologs in barley and/or other cereal species (see Supplementary Table S1 at *JXB* online). Since wheat is a hexaploid, gene-specific primers (Supplementary Table S2) were designed from the conserved coding regions of the three homeologs of each target gene using Primer 3 software. Specificity of the primers and amplification efficiency of the PCRs (Supplementary Table S2) were evaluated as described previously (Mukherjee *et al.*, 2015). Primer information for GA and ABA metabolism and signaling, and *EXPA* and *Taβactin* genes was described previously (Izydorczyk *et al.*, 2018; Han *et al.*, 2019).

### RNA extraction and quantitative RT–PCR

Total RNA extraction, digestion of the RNA samples with DNase, cDNA synthesis and dilution, and subsequent quantitative PCR assays (in duplicate) were performed as described previously (Izydorczyk *et al.*, 2018). The relative transcript levels of target genes were calculated, after normalization with the reference gene *Taβactin*, as described previously (Livak and Schmittgen, 2001). Analysis with NormFinder software (Andersen *et al.*, 2004) revealed *Taβactin* as the most stably expressed reference gene out of three candidate reference genes (*Taβactin*, *TaβTUB*, and *TaCDCP*) analyzed.

### Enzyme extractions and assays

Frozen endosperm samples were ground into fine powder in liquid nitrogen using a pre-chilled mortar and pestle. Extraction and assays for AMY and BAM were performed using the Ceralpha method (K-CERA kit, Megazyme International Ltd, Wicklow, Ireland) and the Betamyl-3 method (K-BETA3 kit, Megazyme International Ltd), respectively. Extraction and assay for α-glucosidase was performed as described previously (Sun and Henson, 1990) with minor modification. Briefly, the fine powder of each sample was homogenized with extraction buffer containing 50 mM sodium phosphate (pH 9.0), 1 M NaCl, and 1% Triton X-100. The homogenates were centrifuged at 6000 *g* for 10 min at room temperature. The extract (0.1 ml) was mixed with 1 ml of the substrate, which was prepared by dissolving 4-nitrophenyl α-d-glucopyranoside (Sigma-Aldrich) in 50 mM sodium acetate (pH 4.6), and then incubated in a water bath at 37 °C for 30 min. After stopping the reaction by adding 0.1 ml of 1 M NaOH, absorbance was determined at 420 nm. Assays for each sample were performed in duplicate.

### Starch content measurements

Freeze-dried endosperm samples were ground to fine powder by a ball mill, and ~100 mg of the fine powder was mixed with 5 ml of ethanol (80%, v/v). The mixture was incubated in a water bath at ~80–85 °C for 5 min followed by addition of another 5 ml of ethanol (80%, v/v) and then centrifugation at 1800 *g* for 10 min. After removing the supernatant, the pellet was re-suspended in 10 ml of 80% ethanol and then centrifuged at 1800 *g* for 10 min. The pellet, after discarding the supernatant, was used for measuring total starch content using the amyloglucosidase–α-amylase method (K-TSTA kit, Megazyme International Ltd). Assays for each sample were performed in duplicate.

### Analysis of sugar contents

Fine powder (~100 mg) of freeze-dried endosperm samples was mixed with 1 ml of 80% ethanol, and the mixture was immediately placed in a water bath at 70–75 °C for 10 min with constant mixing. After centrifugation at 3500 *g* for 10 min, the supernatant was transferred to a fresh tube. The pellet was re-suspended with 1 ml of 80% ethanol and incubated in a water bath at 70–75 °C for 10 min followed by centrifugation at 3500 *g* for 10 min, and these steps were repeated a second time. The supernatant from the three washes was pooled and completely dried under a stream of nitrogen gas. The residue was then mixed with 2 ml of deionized water followed by incubation in boiling water for 5 min. After filtering through a 0.45 µm GH Polypro (GHP) acrodisc syringe filter (Pall Corporation, Port Washington, NY, USA), the sample was analyzed with high-performance anion exchange chromatography (Dionex-ICS-5000; Dionex, Sunnyvale, CA, USA) equipped with a pulsed amperometric detector. Separation of soluble sugars (sucrose, maltose, glucose, and fructose) in the sample was performed with a CarboPac PA-1 column (4×250 mm i.d., Dionex) and a CarboPac PA-1 guard column (4×50 mm i.d., Dionex) at 30 °C using 100 mM NaOH as eluent A and 100 mM NaOH containing 400 mM sodium acetate as eluent B. A sample (10 µl) was injected and eluted at a flow rate of 1 ml min^–1^ with a linear gradient of 50% eluent B for 30 min followed by 100% eluent B for 1 min for washing and 5% eluent B for 15 min for equilibration. The sugars were identified and quantified relative to known standards. Data collection and peak analysis were performed using the Chromeleon 7.1 (Dionex) software.

### Measurement of ABA and GA contents

Extraction of ABA and GA from the different tissues of dry and imbibing seeds and seedlings, and subsequent analysis of their levels by LC-ESI-MS/MS (Agilent 1260-6430; Agilent, Santa Clara, CA, USA) was performed exactly as described previously (Son *et al.*, 2016; Izydorczyk *et al.*, 2018).

### Statistical analysis

Statistically significant differences between samples were determined using Student’s *t*-test at a probability value of *P*<0.05.

## Results

### Seed germination and seedling growth in response to inhibition of ethylene synthesis

**Fig. 1.**
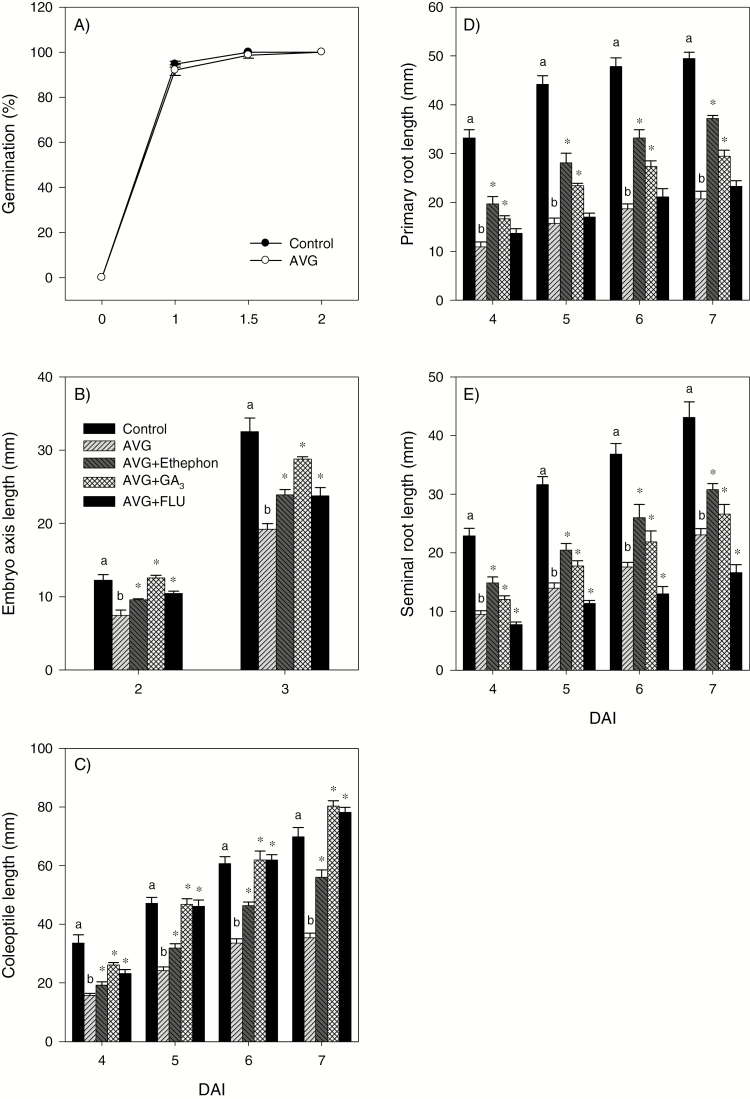
Effects of treatment with an ethylene biosynthesis inhibitor, ethephon, gibberellin, or an abscisic acid biosynthesis inhibitor on germination and seedling growth. Germination (A), and lengths of embryo axis (B), coleoptile (C), primary root (D), and seminal root (E) in response to seed imbibition with the ET biosynthesis inhibitor, aminoethoxyvinylglycine (AVG, 1 mM), AVG (1 mM)+ethephon (10 μM), AVG (1 mM)+GA_3_ (50 μM), or AVG (1 mM)+the ABA biosynthesis inhibitor, fluridone (FLU, 50 μM). Data are means ±SE, *n*=3, where *n* represents a batch of 20 seeds. Different letters indicate statistically significant differences between control and AVG-treated samples, while asterisks represent statistically significant differences between AVG treatment and treatment with AVG+ethephon, AVG+GA_3_, or AVG+FLU (*P*<0.05; Student’s *t*-test). DAI, day(s) after imbibition.

**Fig. 2.**
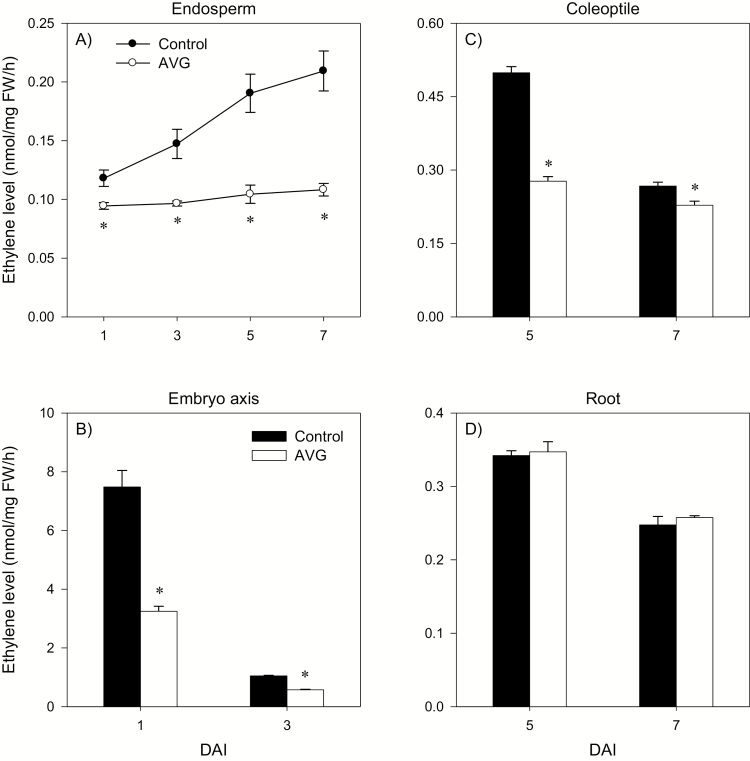
Ethylene levels in different seedling tissues. ET level in the endosperm (A), embryo axis (B), coleoptile (C), and root (D) in response to seed imbibition with AVG (1 mM). Data are means ±SE, *n*=3, where *n* represents a batch of eight tissues (for endosperm, embryo axis at 3 DAI, coleoptile, or root) or a batch of 20 tissues (for embryo axis at 1 DAI). Asterisks indicate statistically significant differences in ethylene levels between control and AVG-treated samples (*P*<0.05; Student’s *t*-test). DAI, day(s) after imbibition.

Treatment with AVG did not affect radicle protrusion, but reduced embryo axis, coleoptile, and primary and seminal root growth (Fig. 1). We detected ET production in all tissues studied; however, the embryo axis, especially at 1 DAI, produced much more ET than the other tissues (Fig. 2). AVG treatment reduced the ET level in all tissues with the exception of the root (Fig. 2). The inhibitory effect of AVG on embryo axis, coleoptile, and root growth was reversed by treatment with ethephon or GA_3_, or fluridone, except that the fluridone treatment either did not have any effect or caused further inhibition of primary and seminal root growth, respectively (Fig. 1B–E). The reversal of the AVG effect on growth by ethephon was partial as only 10 µM was used in order to avoid its inhibitory effect on root growth. Treatment of control seeds with GA_3_ increased embryo axis (2–3 DAI) and coleoptile (5–7 DAI) growth, but did not affect primary/seminal root growth except at 4 DAI (Supplementary Fig. S1). Fluridone also increased coleoptile growth (5–7 DAI) but repressed root growth. Treatment with ethephon (10 μM) did not affect growth in all tissues.

### Transcriptional regulation of endospermic starch degradation

To determine if inhibition of ET production in the endosperm affects storage starch degradation, we examined the expression patterns of starch-degrading genes and activity of the corresponding enzymes, and starch and soluble sugars levels in the endosperm of imbibing seeds in response to AVG treatment.

#### Expression of α-amylase genes in the endosperm

**Fig. 3.**
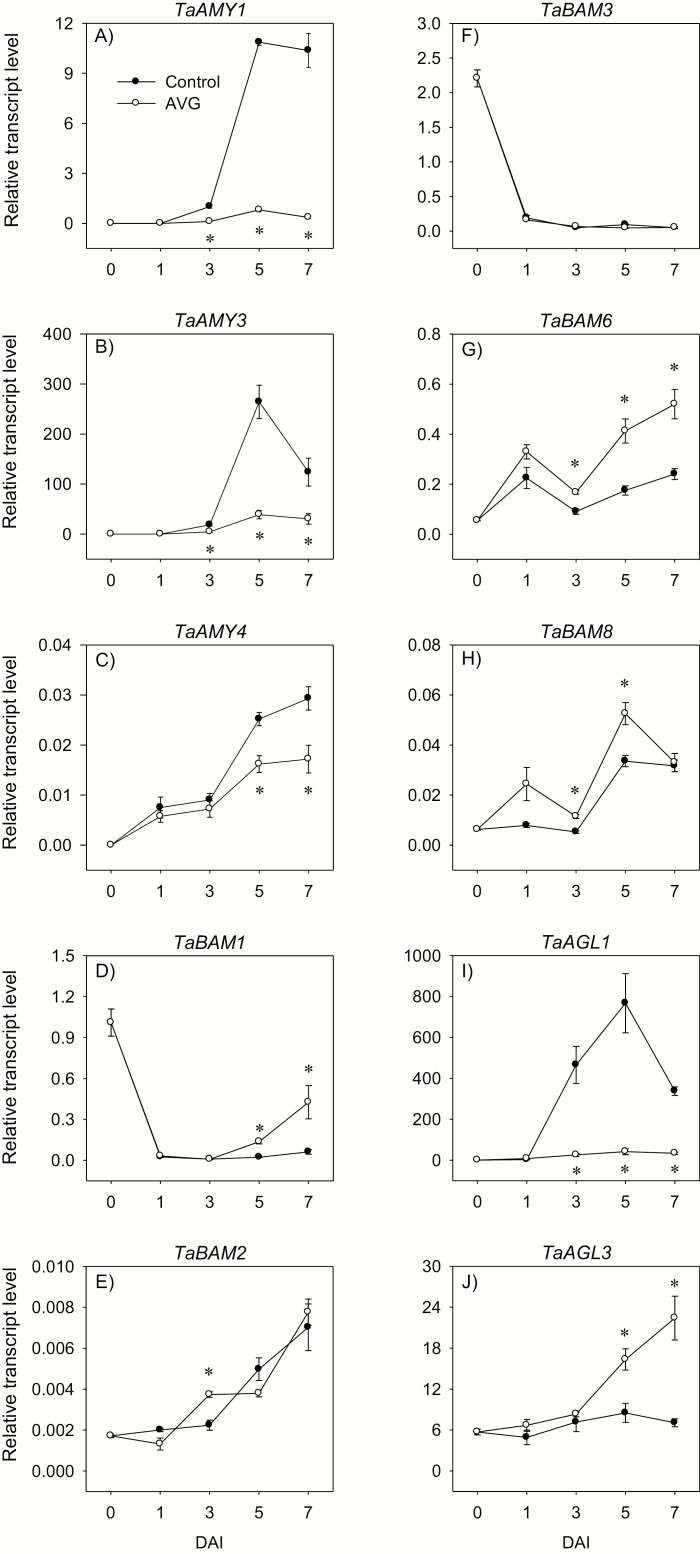
Expression patterns of starch-degrading genes in the endosperm during imbibition. Relative transcript levels of *TaAMY* (A–C), *TaBAM* (D–H), and *TaAGL* (I, J) genes in AVG-treated samples and their respective controls. Transcript levels of *TaAMY*, *TaBAM*, and *TaAGL* genes were determined using *Taβ-actin* as a reference gene and expressed relative to the transcript levels of *TaAMY1* in the control endosperm at 3 DAI, and the transcript levels of *TaBAM1* and *TaAGL1* in the control endosperm at 0 DAI, respectively, which were set to a value of 1. Data are means of three biological replicates ±SE. Asterisks indicate statistically significant differences in expression levels between control and AVG-treated samples (*P*<0.05; Student’s *t*-test). DAI, day(s) after imbibition.

No or a minimal expression level of *TaAMY* genes was detected in the endosperm of dry seeds; however, their expression levels increased with imbibition in both control and AVG-treated seeds (Fig. 3A–C); *TaAMY3* showed the highest magnitude of expression followed by *TaAMY1* and then *TaAMY4*. However, AVG treatment decreased the expression levels of *TaAMY* genes; *TaAMY1* (>9-fold) and *TaAMY3* (>4-fold) following 3 DAI, and *TaAMY4* (>1.6-fold) following 5 DAI.

#### Expression of β-amylase genes in the endosperm

The transcripts of all *TaBAM* genes were detected in the endosperm of dry seeds (Fig. 3D–H). High transcript levels of *TaBAM1* and *TaBAM3* were observed in dry seeds, and their transcript levels decreased substantially by 1 DAI in both AVG-treated and control samples, and remained at low levels afterwards. In contrast, the expression levels of *TaBAM2*, *TaBAM6*, and *TaBAM8* exhibited gradual increases with imbibition in both AVG-treated and control seeds. AVG treatment did not affect the expression levels of *TaBAM2* and *TaBAM3* but up-regulated *TaBAM1*, *TaBAM6*, and *TaBAM8* following 3 or 5 DAI (1.6- to 7-fold) (Fig. 3D, G, H).

#### Expression of α-glucosidase genes in the endosperm

The transcripts of *TaAGL* genes (*TaAGL1* and *TaAGL3*) were detected in the endosperm of dry seeds (Fig. 3I, J). In the control samples, *TaAGL1* expression showed a substantial increase with imbibition, resulting in a much higher level of expression than that observed for *TaAGL3* (up to 90-fold). However, AVG treatment repressed *TaAGL1* expression (>10-fold) following 3 DAI. Almost a similar level of *TaAGL3* expression was maintained during the entire imbibition period, and AVG treatment enhanced its expression level (2- to 3-fold) following 5 DAI (Fig. 3J).

### Activities of starch-degrading enzymes in the endosperm

**Fig. 4.**
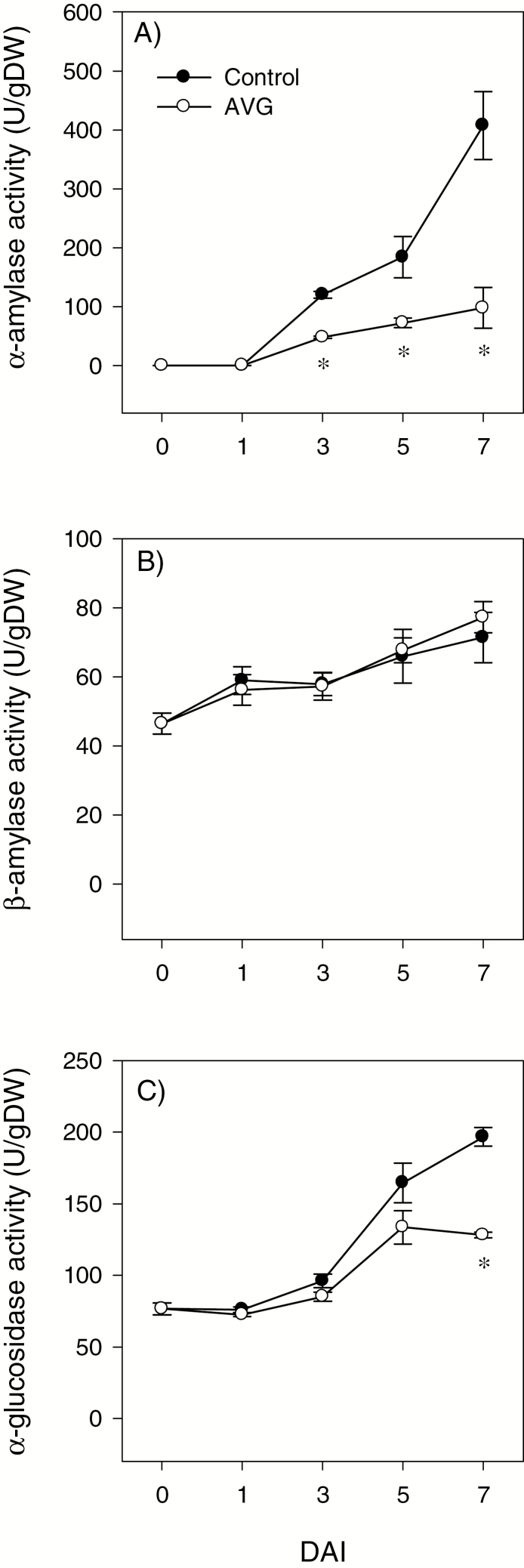
Activities of starch-degrading enzymes in the endosperm during imbibition. Activities of α-amylase (A), β-amylase (B), and α-glucosidase (C) in AVG-treated samples and their respective controls. Data are means of three biological replicates ±SE. Asterisks indicate statistically significant differences in enzyme activities between control and AVG-treated samples (*P*<0.05; Student’s *t*-test). DAI, day(s) after imbibition.

The activities of AMY, BAM, and AGL were detected in the endosperm of dry seeds (Fig. 4). Although AMY activity increased with imbibition, it was inhibited by AVG treatment (>2-fold) following 1 DAI (Fig. 4A). The BAM activity detected in the endosperm of dry seeds showed a slight increase during the entire period studied, and it was not affected by treatment with AVG (Fig. 4B). The activity of AGL detected in the endosperm of dry seeds was also maintained at a similar level until 3 DAI, after which its activity increased (1.5- to 2-fold) in both samples through to the end of the period studied (Fig. 4C). However, AVG treatment repressed the AGL activity at 7 DAI.

### Endospermic dry weight and starch and soluble sugar contents

**Fig. 5.**
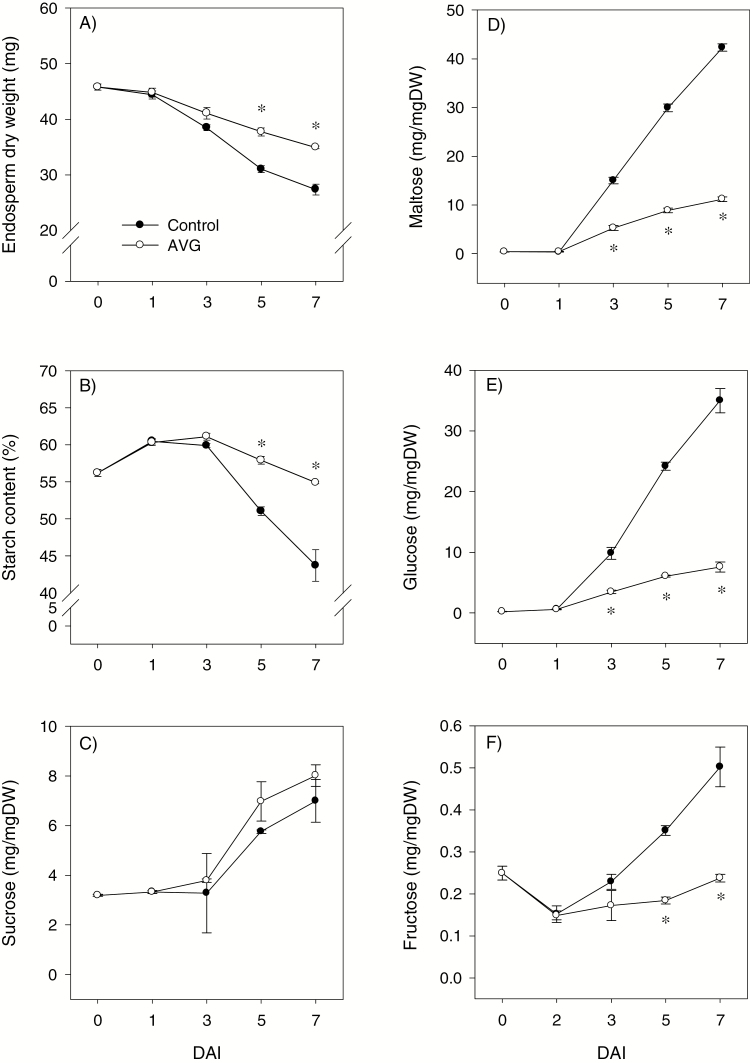
Endosperm dry weight and starch and soluble sugar contents during imbibition. Dry weight (A), and contents of starch (B), sucrose (C), maltose (D), glucose (E), and fructose (F) in AVG-treated samples and their respective controls. Data are means of three biological replicates ±SE. Asterisks indicate statistically significant differences in dry weight and starch and soluble sugar contents between control and AVG-treated samples (*P*<0.05; Student’s *t*-test). DAI, day(s) after imbibition.

Endospermic dry weight declined with imbibition in both control and AVG-treated seeds; however, the AVG-treated seeds exhibited higher dry weight at 5 and 7 DAI than the control seeds (Fig. 5A). Consistent with this result, endospermic starch content exhibited a decrease with imbibition in both seed samples; however, its level was higher in AVG-treated seeds than in the respective controls at 5 and 7 DAI (Fig. 5B). Soluble sugars including maltose, glucose, and fructose were determined in the endosperm of dry seeds, and their level increased (2- to 157-fold) with imbibition (Fig. 5D–F). The levels of maltose and glucose were reduced by AVG treatment following 3 DAI (2.8- to 4.6-fold), while the reduction of fructose level (over ~2-fold) due to AVG treatment was evident only after 5 DAI. The endospermic sucrose level, however, showed a gradual increase with imbibition and it was not affected by AVG treatment (Fig. 5C).

### Transcriptional regulation of ABA/GA balance in the endosperm

To determine if the effects of inhibition of ET synthesis on endospermic starch degradation are associated with changes in ABA/GA balance, we investigated the expression patterns of ABA and GA metabolism and signaling genes, and measured ABA and GA levels in the endosperm of imbibing seeds in response to AVG treatment.

#### Transcriptional regulation of GA metabolism and signaling in the endosperm

**Fig. 6.**
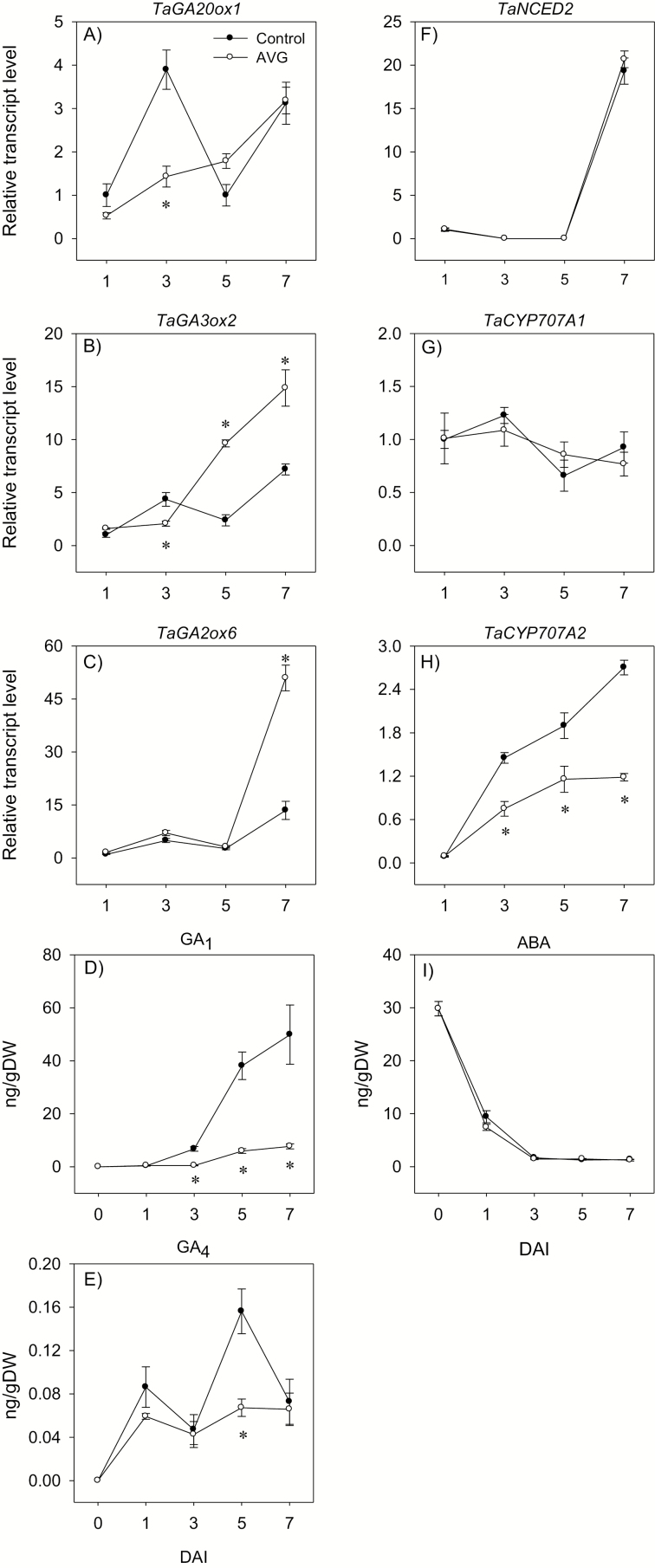
Expression patterns of gibberellin and abscisic acid metabolism genes, and endogenous gibberellin and abscisic acid levels in the endosperm during imbibition. Relative transcript levels of *TaGA20ox1* (A), *TaGA3ox2* (B), *TaGA2ox6* (C), *TaNCED2* (F), and *TaCYP707A* genes (G and H) in AVG-treated samples and their respective controls. Transcript levels were determined exactly as described in Fig. 3 and expressed relative to the transcript levels of *TaGA20ox1*, *TaGA3ox2*, *TaGA2ox6*, *TaNCED2*, and *TaCYP707A1* in the control endosperm at 0 DAI, which were set to a value of 1. Endospermic GA_1_ (D), GA_4_ (E), and ABA (I) levels in AVG-treated samples and their respective controls. Data are means of three biological replicates ±SE except that the GA_1_ data for the control samples at 5 DAI are means of five biological replicates ±SE. Asterisks indicate statistically significant differences in expression levels or hormone contents between control and AVG-treated samples (*P*<0.05; Student’s *t*-test). DAI, day(s) after imbibition.

**Fig. 7.**
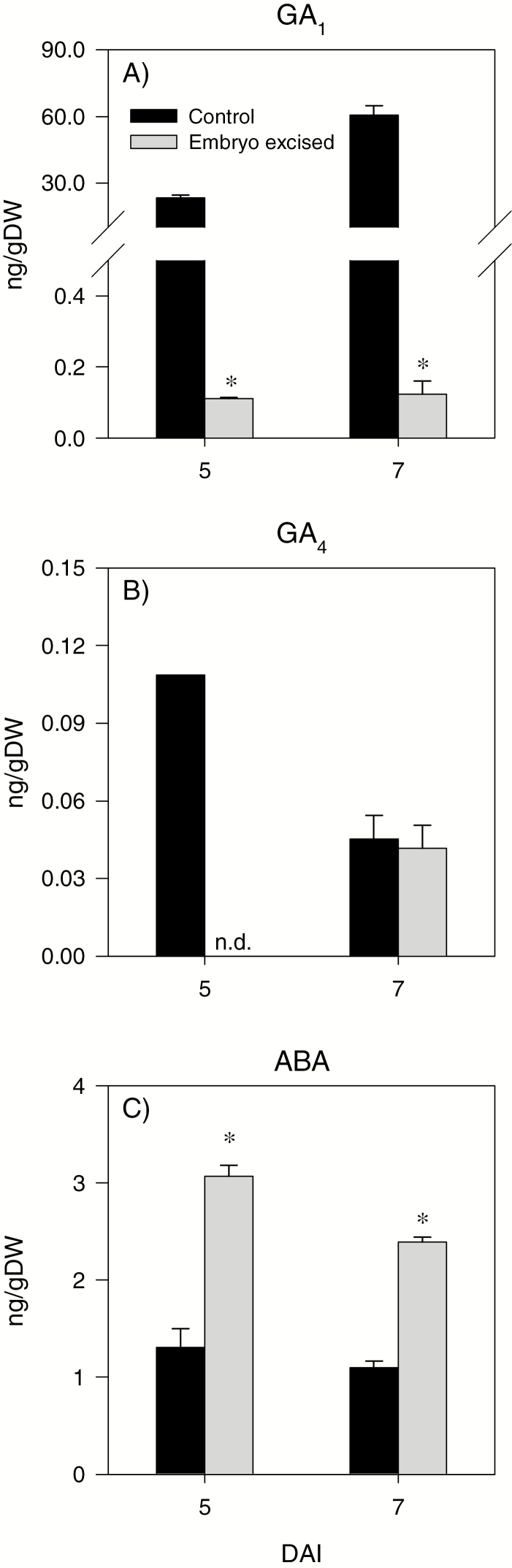
Endospermic gibberellin and abscisic acid levels in response to embryo excision before the start of imbibition. Endospermic GA_1_ (A), GA_4_ (B), and ABA (C) levels of seeds imbibed for 5 d and 7 d with embryo axis (control) and no embryo axis (embryo excised). Data are means of three biological replicates ±SE. Asterisks indicate statistically significant differences in hormone contents between endosperm samples imbibed with and with no embryo (*P*<0.05; Student’s *t*-test). n.d., not detected.

**Fig. 8.**
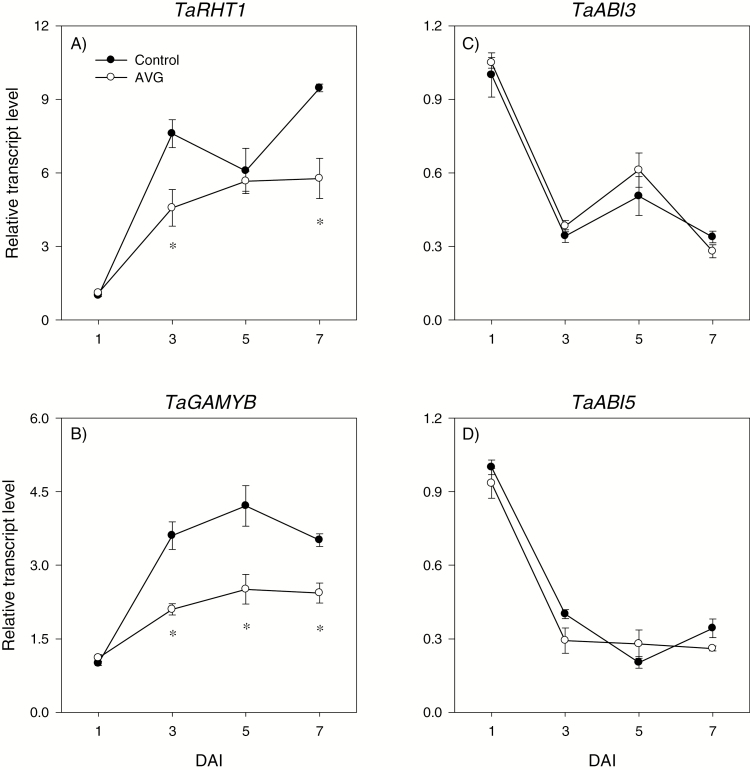
Expression patterns of gibberellin and abscisic acid signaling genes in the endosperm during imbibition. Transcript levels of *TaRHT1* (A), *TaGAMYB* (B), *TaABI3* (C), and *TaABI5* (D) were determined in AVG-treated samples and their respective controls exactly as described in Fig. 3 and expressed relative to their respective transcript levels in the control endosperm at 0 DAI, which were set to a value of 1. Data descriptions are as shown in Fig. 3. DAI, day(s) after imbibition.

A similar expression level of the GA biosynthetic gene *TaGA20ox1* was evident between the endosperms of control and AVG-treated seeds, except for the transient increase observed in the control samples at 3 DAI (Fig. 6A). The expression level of *TaGA3ox2* increased with imbibition in both control and AVG-treated seeds; however, endosperms of AVG-treated seeds exhibited a higher expression level (>2-fold) following 5 DAI (Fig. 6B). Furthermore, AVG treatment led to an ~4-fold induction in the expression level of the GA catabolic gene *GA2ox6* at 7 DAI (Fig. 6C). Bioactive GA_1_ and GA_4_ were detected in the endosperm of imbibed but not dry seeds, and their levels increased with imbibition (Fig. 6D, E). However, AVG treatment caused a substantial reduction in the endospermic GA_1_ level (6.5- to 15-fold) as compared with that observed in the control seeds following 3 DAI (Fig. 6D). The level of GA_4_ was also reduced by AVG treatment (>2-fold) at 5 DAI (Fig. 6E). Embryo axis excision before the start of imbibition led to a marked reduction in the accumulation of endospermic bioactive GA during the later phases of imbibition, at 5 and/or 7 DAI (Fig. 7A, B). The expression level of the endospermic GA signaling gene *TaRHT1*, which encodes the wheat DELLA protein, showed an increase with imbibition in both control and AVG-treated seeds; however, AVG treatment repressed its expression at 3 and 7 DAI (Fig. 8A). The expression level of the other GA signaling gene, *TaGAMYB*, also showed a gradual increase with imbibition in both seed samples (Fig. 8B), but AVG treatment repressed its expression following 3 DAI.

#### Transcriptional regulation of ABA metabolism and signaling in the endosperm

Expression of the ABA biosynthetic gene *TaNCED1* was below the detectable level in the endosperm of imbibing control and AVG-treated seeds (data not shown). Similarly, no or a minimal expression level of *TaNCED2* was detected in both seed samples except that drastic increases in its expression level were observed at 7 DAI (Fig. 6F). The expression level of one of the ABA catabolic genes, *TaCYP707A1*, remained constant during imbibition, and it was not affected by AVG treatment, while that of *TaCYP707A2* increased with imbibition in both seed samples and its expression level was decreased by AVG treatment following 3 DAI (Fig. 6G, H). A high level of ABA was detected in the endosperm of dry seeds, but it decreased to a very low level in both control and AVG-treated seed samples as imbibition progressed, and no effect of AVG treatment was evident (Fig. 6I). Embryo axis excision led to a substantial increase in endospermic ABA levels during the later phases of imbibition, at 5 and 7 DAI (Fig. 7C). The expression levels of endospermic genes encoding the downstream ABA signaling transcription factors, *TaABI3* and *TaABI5*, decreased with imbibition, and there was no apparent effect of AVG on their expression levels (Fig. 8C, D).

### Transcriptional regulation of ABA/GA balance in post-germination seedlings

To determine if the effects of inhibition of ET synthesis on seedling growth are associated with changes in ABA/GA balance in non-nurturing tissues, we investigated the expression patterns of ABA and GA metabolism and signaling genes, and measured ABA and GA levels in the embryo axis, coleoptile, and root tissues in response to AVG treatment.

#### Transcriptional regulation of GA and ABA metabolism and signaling in the embryo axis

**Fig. 9.**
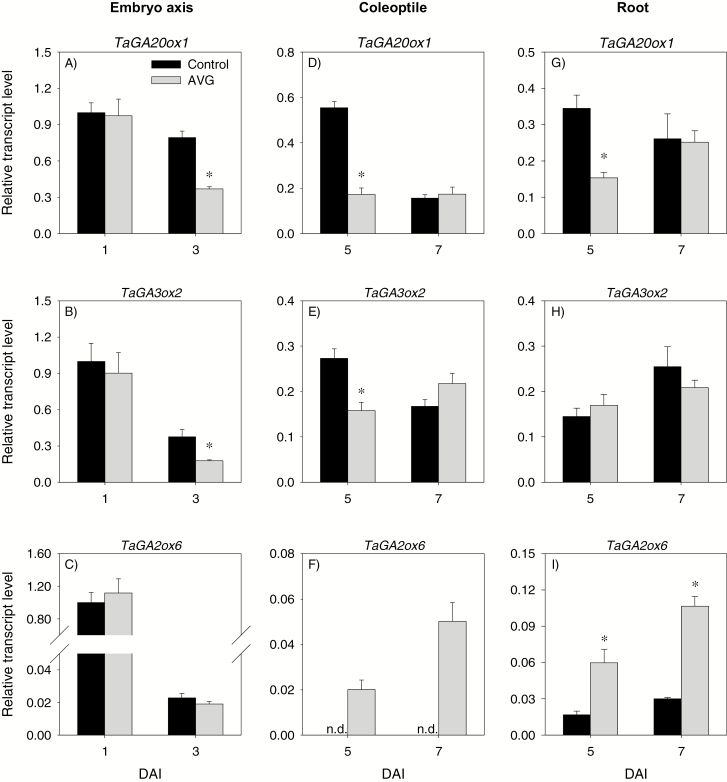
Expression patterns of gibberellin metabolism genes in the embryo axis, coleoptile, and root of post-germination seedlings. Relative transcript levels of *TaGA20ox1* (A, D, and G), *TaGA3ox2* (B, E, and H), and *TaGA2ox6* (C, F, and I) in AVG-treated samples and their respective controls. Transcript levels of each gene were determined exactly as described in Fig. 3 and expressed relative to their respective transcript level in the control embryo axis at 1 DAI, which was set to a value of 1. Data descriptions are as shown in Fig. 3. DAI, day(s) after imbibition; n.d., not detected.

**Fig. 10.**
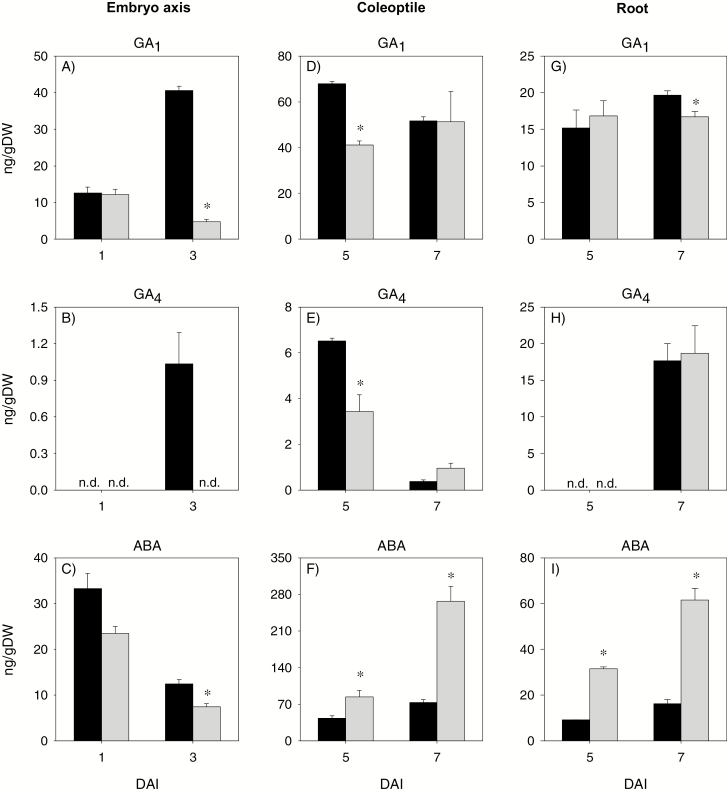
Gibberellin and abscisic acid levels in the embryo axis, coleoptile, and root of post-germination seedlings. GA_1_ (A, D, and G), GA_4_ (B, E, and H), and ABA (C, F, and I) in AVG-treated samples and their respective controls. Data are means of three biological replicates ±SE. Asterisks indicate statistically significant differences in hormone contents between control and AVG-treated samples (*P*<0.05; Student’s *t*-test). DAI, day(s) after imbibition; n.d., not detected.

**Fig. 11.**
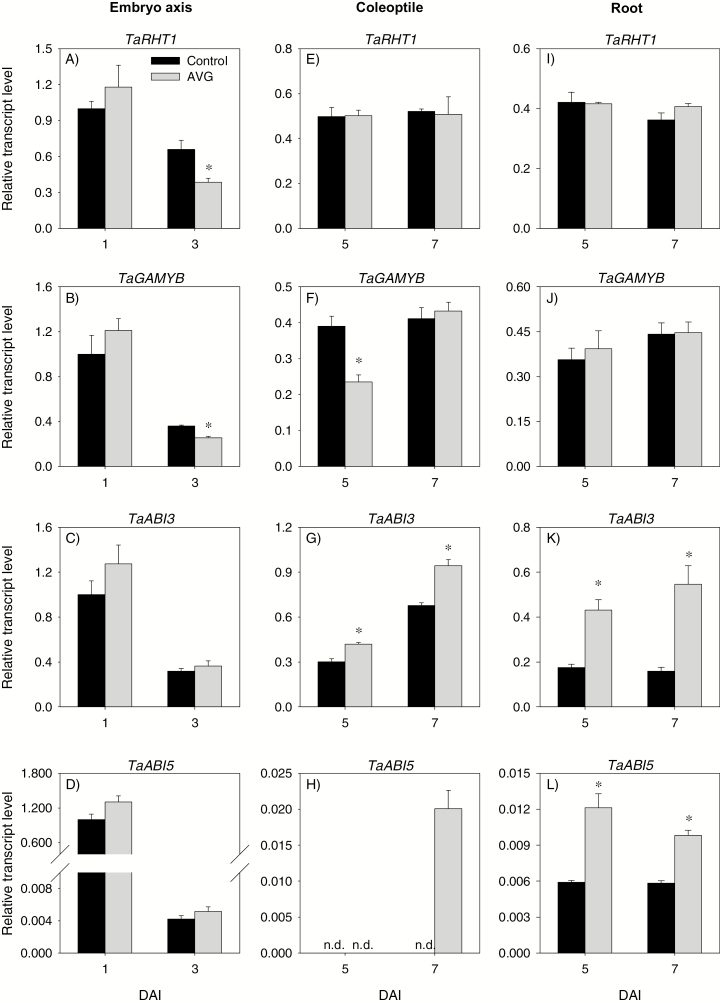
Expression patterns of gibberellin and abscisic acid signaling genes in the embryo axis, coleoptile, and root of post-germination seedlings. Transcript levels of *TaRHT1* (A, E, and I), *TaGAMYB* (B, F, and J), *TaABI3* (C, G, and K), and *TaABI5* (D, H, and L) were determined in AVG-treated samples and their respective controls exactly as described in Fig. 3 and expressed relative to their respective transcript levels in the control embryo axis at 1 DAI, which were set to a value of 1. Data descriptions are as shown in Fig. 3. DAI, day(s) after imbibition; n.d., not detected.

There was no differential expression of GA biosynthetic (*TaGA20ox1* and *TaGA3ox2*) and catabolic (*TaGA2ox6*) genes between the control and AVG-treated embryo axis at 1 DAI (Fig. 9A–C). However, AVG treatment decreased (>2-fold) the expression levels of *TaGA20ox1* and *TaGA3ox2* in the embryo axis at 3 DAI with no effect on that of *TaGA2ox6*. Consistently, the level of GA_1_ in 1 DAI embryo axis was not affected by AVG treatment, while its level in the 3 DAI embryo axis exhibited a substantial decrease (8.5-fold) (Fig. 10A). The other bioactive GA, GA_4_, was also detected in 3 DAI embryo axis, but its level decreased to an undetectable level due to AVG treatment (Fig. 10B). No effect of AVG treatment was evident in the expression levels of *TaRHT1* and *TaGAMYB* in 1 DAI embryo axis; however, AVG treatment decreased the expression levels of both genes in the 3 DAI embryo axis tissue (Fig. 11A, B).

**Fig. 12.**
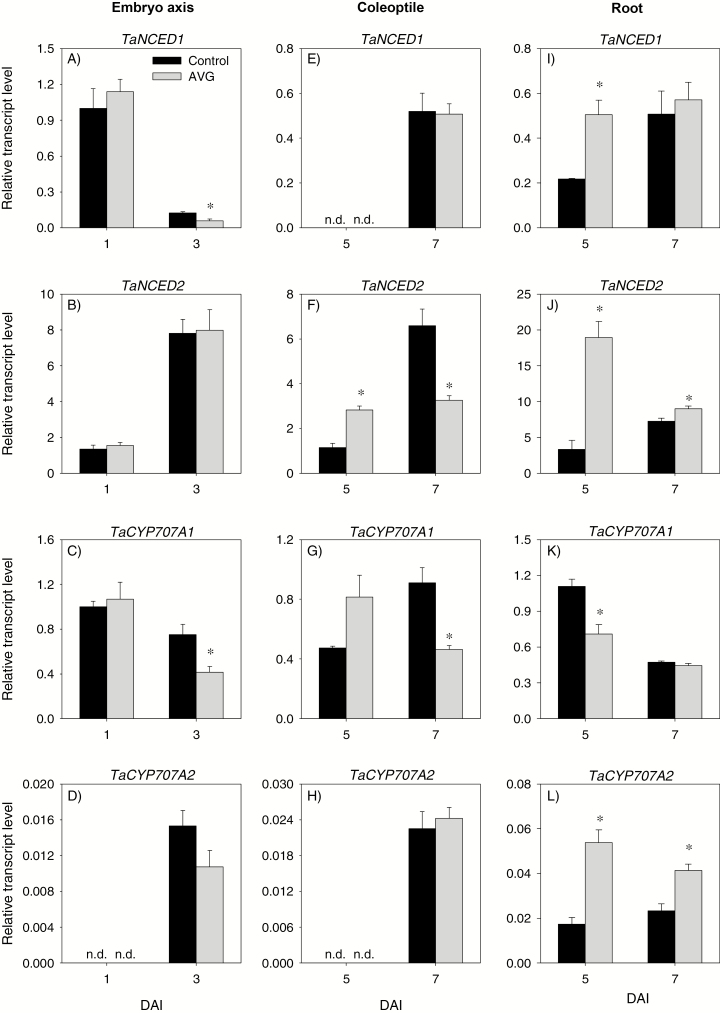
Expression patterns of abscisic acid metabolism genes in the embryo axis, coleoptile, and root of post-germination seedlings. Relative transcript levels of *TaNCED1* (A, E, and I), *TaNCED2* (B, F, and J), *TaCYP707A1* (C, G, and K), and *TaCYP707A2* (D, H, and L) in AVG-treated samples and their respective controls. Transcript levels of *NCED* and *CYP707A* genes were determined exactly as described in Fig. 3 and expressed relative to *TaNCED1* and *TaCYP707A1* transcript levels in the control embryo axis at 1 DAI, respectively, which were set to a value of 1. Data descriptions are as shown in Fig. 3. DAI, day(s) after imbibition; n.d., not detected.

The expression levels of all ABA metabolism genes in 1 DAI embryo axis were unaffected by AVG treatment (Fig. 12A–D), leading to no change in ABA level (Fig. 10C). In contrast, AVG treatment led to reductions in the expression levels of *TaNCED1* and *TaCYP707A1* genes, and the ABA level in 3 DAI embryo axis. AVG treatment did not affect the expression levels of ABA signaling genes, *TaABI3* and *TaABI5*, in the embryo axis at either time point (Fig. 11C, D).

#### Transcriptional regulation of GA and ABA metabolism and signaling in the coleoptile

Treatment with AVG repressed the expression levels of *TaGA20ox1* and *TaGA3ox1* in the coleoptile at 5 DAI but increased that of *TaGA2ox6*, leading to reduction of GA_1_ and GA_4_ levels (Figs 9D–F, 10D, E). However, no effect of AVG treatment was evident on the expression levels of *TaGA20ox1* and *TaGA3ox1*, and bioactive GA levels in the coleoptile at 7 DAI although up-regulation of *TaGA2ox6* was evident. AVG treatment did not affect the expression levels of *TaRHT1* in the coleoptile at either time point; however, it repressed that of *TaGAMYB* at 5 DAI (Fig. 11E, F).

With respect to ABA metabolism genes, treatment with AVG enhanced the expression level of *TaNCED2* in the coleoptile at 5 DAI, with no effect on the expression levels of the other genes (Fig. 12E–H). Consistently, AVG treatment led to an increase in ABA level (Fig. 10F). The AVG treatment, however, caused reduction in the expression levels of *TaNCED2* (2-fold) and *TaCYP707A1* (2-fold) in the coleoptile at 7 DAI (Fig. 12F, G), and this effect was associated with an increase in ABA level (>3-fold) (Fig. 10F). Treatment with AVG led to an enhanced expression level of *TaABI3* at both time points and of *TaABI5* at 7 DAI (Fig. 11G, H).

#### Transcriptional regulation of GA and ABA metabolism and signaling in the root

The expression levels of *TaGA20ox1* and *TaGA3ox1* in the root were not affected by AVG treatment except that the expression of *TaGA20ox1* was repressed at 5 DAI (Fig. 9G, H). The treatment, however, enhanced the expression level of *TaGA2ox6* (>3-fold) at 5 and 7 DAI (Fig. 9I). Despite these results, AVG treatment did not affect root GA_1_ or GA_4_ levels except the slight reduction observed in GA_1_ level at 7 DAI (Fig. 10G, H). Treatment with AVG did not have an effect on the expression levels of root *TaRHT1* and *TaGAMYB* at either time point (Fig. 11I, J).

Treatment with AVG enhanced the expression levels of root *TaNCED1* at 5 DAI (>2-fold), and of *TaNCED2* (1.2- to 5.7-fold) and *TaCYP707A2* (1.8- to 3.1-fold) at both 5 and 7 DAI, but repressed that of *TaCYP707A1* at 5 DAI (Fig. 12I–L). Root ABA level at 5 and 7 DAI exhibited an increase (>3-fold) in response to AVG treatment (Fig. 10I). The AVG treatment also enhanced the expression levels of root *TaABI3* and *TaABI5* genes (1.7- to 3.4-fold) at both 5 and 7 DAI (Fig. 11K, L).

#### Expression analysis of cell elongation genes

**Fig. 13.**
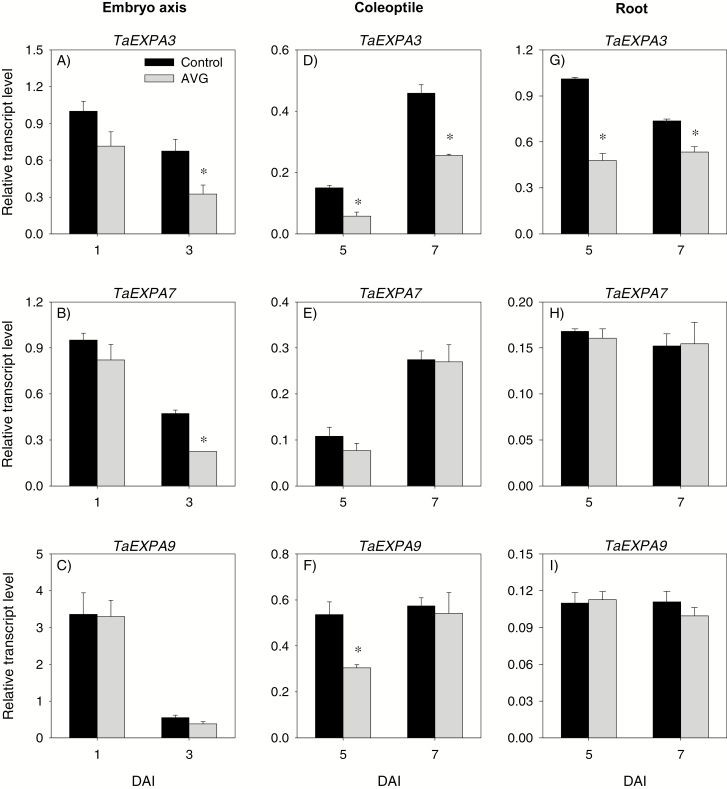
Expression patterns of *α*-*expansin* genes in the embryo axis, coleoptile, and root of post-germination seedlings. Transcript levels of *TaEXPA3* (A, D, and G), *TaEXPA7* (B, E, and H), and *TaEXPA9* (C, F, and I) were determined in AVG-treated samples and their respective controls exactly as described in Fig. 3 and expressed relative to the transcript level of *TaEXPA3* in the control embryo axis at 1 DAI, which was set to a value of 1. Data descriptions are as shown in Fig. 3. DAI, day(s) after imbibition.

To determine if the effect of the reduced ET level on seedling growth is mediated by transcriptional regulation of cell elongation, we monitored the expression patterns of *TaEXPA* genes. Our analysis showed reduction in the expression level of *TaEXPA3* in the embryo axis at 3 DAI, and in the coleoptile and root tissues at both 5 and 7 DAI in response to AVG treatment (Fig. 13A, D, G). The other two *TaEXPA* genes, *TaEXPA7* and *TaEXPA9*, showed AVG-mediated down-regulation only in 3 DAI embryo axis and 5 DAI coleoptile, respectively (Fig. 13B, F).

## Discussion

Seed germination and seedling growth are regulated mainly by GA and ABA; however, other plant hormones such as ET also control these early developmental processes. This study investigated the molecular mechanisms underlying the role of ET in regulating ABA/GA balance, and thereby seed germination, and starch degradation and seedling growth in wheat. Although ET promotes seed germination in dicots (Petruzzelli *et al.*, 2000; Matilla and Matilla-Vázquez, 2008; Linkies *et al.*, 2009), inhibition of its synthesis did not affect radicle protrusion in wheat (Fig. 1A) as observed in other monocots such as barley and rice (Locke *et al.*, 2000; Gianinetti *et al.*, 2007). Mobilization of endospermic starch is required to provide energy for seedling growth in cereals (Zeeman *et al.*, 2010), and the expression of starch-degrading genes such as *AMYs* and activity of the corresponding enzymes are reported to increase with seed imbibition (Perata *et al.*, 1992; Guglielminetti *et al.*, 1995; Sun *et al.*, 2018). Our data revealed that inhibition of ET synthesis during imbibition represses the expression of endospermic *TaAMY* genes (*TaAMY1*, *TaAMY3*, and *TaAMY4*) and the *TaAGL1* gene, together with the activity of AMY and AGL, leading to inhibition of storage starch degradation and reduction in the levels of soluble sugars including maltose, glucose, and fructose (Figs 3–5). Since previous studies implicated ET in the regulation of seedling growth (Matilla 2000; Yin *et al.*, 2017), the association of these effects of reduced endospermic ET level with decreases in embryo axis, coleoptile, and root growth (Fig. 1B–E) indicate that ET regulates seedling growth in wheat partly through transcriptional control of storage starch degradation. Inhibition of ET synthesis; however, did not alter BAM activity despite causing up-regulation of specific *TaBAM* genes (*TaBAM1*, *TaBAM6*, and *TaBAM8*) (Figs 3, 4), indicating that these genes are regulated post-transcriptionally. The concomitant gradual increases in the levels of their expression during imbibition with that of BAM activity are indicative of the involvement of *TaBAM6* and *TaBAM8*, and to a much lesser extent *TaBAM2*, in storage starch degradation. However, the activity of BAM during imbibition was markedly lower than that of AMY, indicating its minor role in storage starch degradation (Fig. 4A, B). Consistently, mutant seeds deficient in BAM activity can complete their germination and produce normal seedlings (Kihara *et al.*, 1999; Kaneko *et al.*, 2000).

GA and ABA act as primary regulators of starch degradation in cereal seeds through their antagonistic action (Gubler *et al.*, 1995; Gómez-Cadenas *et al*., 1999, 2001), and ET has been reported to interact with GA and ABA in other plant systems (Kende *et al.*, 1998; Choi, 2007). In accordance with our recent report (Izydorczyk *et al.*, 2018), no bioactive GA but a high level of ABA was detected in the endosperm of dry seeds, and the level of ABA decreased to a very low level during germination (Fig. 6D, E, I). Such changes in GA and ABA levels have also been reported either in the embryo axis or at the whole-seed level during seed germination in wheat and other species (Ogawa *et al.*, 2003; Gubler *et al.*, 2008; Izydorczyk *et al.*, 2018). Endospermic bioactive GAs were detected only after completion of germination, which was determined by the emergence of coleorhiza through the seed coat, and their levels increased with imbibition (Fig. 6D, E). Inhibition of ET synthesis during imbibition did not affect either the endospermic ABA level or the expression levels of ABA signaling genes *TaABI3* and *TaABI5* (Figs 6I, 8C, D); however, it decreased bioactive GA levels and GA sensitivity, as evidenced by down-regulation of *TaGAMYB* (Figs 6, 8B). These results along with the promotion of embryo axis and/or coleoptile growth, which is fueled through mobilization of storage starch, by GA_3_, or by fluridone (Supplementary Fig. S1) indicate the significance of endospermic GA level and sensitivity in mediating ET-induced modulation of the ABA/GA balance and thereby transcriptional regulation of starch degradation. In agreement with this, inhibition of ET synthesis alters the expression patterns of GA biosynthetic genes, *TaGA20ox2* and *TaGA3ox2*, and thereby represses seed germination (Iglesias-Fernández and Matilla, 2010). The up-regulation of *TaGA3ox2* in the endosperm where bioactive GA levels were reduced due to inhibition of ET synthesis (Fig. 6B) shows its negative feedback regulation by GA as observed for specific *GA3ox* genes in Arabidopsis (Matsushita *et al.*, 2007), while the repression of endospermic *TaRHT1* (Fig. 8A), which encodes wheat DELLA protein that acts as a negative regulator of GA signaling, suggests either its post-transcriptional regulation or the occurrence of GA signaling through a DELLA-independent pathway (Cao *et al.*, 2006).

To gain insights into the origins of ABA and GA detected in the endosperm, we performed experiments involving embryo axis excision prior to imbibition. Detection of GA_1_ and GA_4_ in the endosperm samples imbibed with no embryo axis (Fig. 7A, B) is indicative of their synthesis in the aleurone, while the substantial reduction of their levels, especially that of GA_1_, as compared with the control (imbibed with embryos) implicates that GA transport from non-endospermic tissues accounts for the majority of the bioactive GA detected in the endosperm, and thereby regulation of storage starch degradation. Our results are consistent with a report that implicated the scutellum/embryo axis as the main site of GA synthesis in germinating wheat seeds (Appleford and Lenton, 1997). In contrast, Kaneko *et al.* (2003) suggested that no GA synthesis occurs in the aleurone of germinating rice seeds. However, their conclusion was merely based on expression analysis of GA biosynthesis genes. Embryo axis excision prior to imbibition, on the other hand, increased the endospermic ABA level (Fig. 7C), showing that most of the ABA accumulated in the endosperm is synthesized *in situ*, and this induction of the ABA level is consistent with a reduction in the levels of GA, which regulates ABA synthesis negatively (Seo *et al.*, 2009).

ET acts as a positive regulator of shoot growth in cereal seedlings (Furukawa *et al.*, 1997; Locke *et al.*, 2000; Yin *et al.*, 2017). Consistently, inhibition of ET synthesis caused decreases in embryo axis and coleoptile growth, and these effects were reversed by treatments with ethephon or exogenous GA or fluridone (Fig. 1B, C). While treatment with GA_3_ or fluridone fully reversed coleoptile growth, ethephon caused only partial reversal and this result, along with the absence of any effect of ethephon on coleoptile growth in the control samples (Supplementary Fig. S1), indicates that the ethephon concentration we used was not sufficient to increase ET to the level where it promotes further coleoptile growth. It is well documented that GA promotes growth via degrading the growth-repressing DELLA proteins (Olszewski *et al.*, 2002; Achard *et al.*, 2009) and thereby activating expansins (Cho and Cosgrove, 2004), whereas ABA inhibits growth via repressing cell wall extensibility (da Silva *et al.*, 2008). Previous studies in other plant systems have shown that ET regulates growth via modulating the balance between these two hormones (Kende *et al.*, 1998; Choi, 2007). In the present study, inhibition of ET synthesis did not affect the expression levels of ABA and GA metabolism and signaling genes, and ABA and GA levels in the embryo axis at the early stage (1 DAI) (Figs 9–12). However, it caused reductions in the ABA level, potentially through repression of *TaNCED1*, with no effect on ABA sensitivity, as evidenced by the expression patterns of *ABI3* and *ABI5*, at the later stage (3 DAI) (Figs 10–12). Despite these results, repression of *TaEXPA3* and *TaEXPA7* and inhibition of embryo axis growth (Figs 1B, 13A, B) were evident. These results, along with the prevalence of reduced bioactive GA levels and sensitivity, mainly through repressions of *TaGA20ox1* and *TaGA3ox2*, and *TaGAMYB*, respectively (Figs 9–11), highlight that ET-mediated transcriptional regulation of GA biosynthesis and signaling modulates ABA/GA balance, and thereby cell wall expansion and embryo axis growth.

With respect to the coleoptiles, reduction of the ET level at 5 DAI caused a decrease in bioactive GA levels and sensitivity via altering the expression levels of GA metabolism genes and repressing *TaGAMYB* while enhancing ABA content and sensitivity, mainly through up-regulation of *TaNCED2* and *TaABI3*, respectively (Figs 9–12). These results together with the repression of *TaEXPA3* and *TaEXPA9* and reduction of coleoptile growth (Figs 1C, 13D, F) demonstrate that transcriptional control of GA and ABA metabolism and signaling by ET acts as a primary regulator of ABA/GA balance, and thereby cell wall expansion and coleoptile growth. However, as the coleoptile grew further, inhibition of ET synthesis increased the ABA level and sensitivity, mainly through down-regulating *TaCYP707A1* and up-regulating *TaABI3* and *TaABI5*, respectively, with no effects on bioactive GA levels and expression of GA signaling genes. These observations along with repression of *TaEXPA3* indicate the significance of transcriptional regulation of ABA catabolism and signaling in mediating ET-induced modulation of ABA/GA balance, and thereby cell wall expansion and coleoptile elongation at the later seedling growth stage.

Both exogenous ET and inhibition of ET synthesis have been reported to repress radicle/root growth in rice seedlings (Gianinetti *et al.*, 2007; Ma *et al.*, 2014). Likewise, inhibition of ET synthesis caused reduction in root growth (Fig. 1D, E). This effect was reversed by treatment with ethephon or GA but not by fluridone, which reportedly represses root elongation (Supplementary Fig. S1, Spollen *et al.*, 2000; Han *et al.*, 2004). The detection of a higher ABA level in AVG-treated roots despite the absence of any change in root ET level (Figs 2D, 10I) indicates that the root ABA level is regulated by ET released from the embryo axis and/or a portion of the ABA detected in the root is transported from the coleoptile, where ABA accumulation was evident (Fig. 10F). The enhancement of the ABA level in AVG-treated roots along with up-regulation of *TaABI3* and *TaABI5* also explains the transcriptional induction of *TaNCED* genes and *TaCYP707A2* as these genes are under positive feedback and feedforward regulation by ABA, respectively (Xiong and Zhu, 2003; Saito *et al.*, 2004). ABA accumulation in AVG-treated roots at the later stage of seedling growth was associated with up-regulation of *TaGA2ox6* and reduction of the GA_1_ level (Figs 9I, 10G), reflecting the negative regulation of the GA level by ABA (Seo *et al.*, 2009). However, AVG treatment did not alter the expression levels of *TaRHT1* and *TaGAMYB* in the root despite the repression of *TaEXPA3* (Figs 11, 13). This result, along with the absence of any effect of exogenous GA on root growth in the control samples (Supplementary Fig. S1), indicates that ET-mediated regulation of ABA metabolism in the coleoptile and ABA signaling in the root determine the ABA/GA balance and thereby cell wall expansion and root growth.

**Fig. 14.**
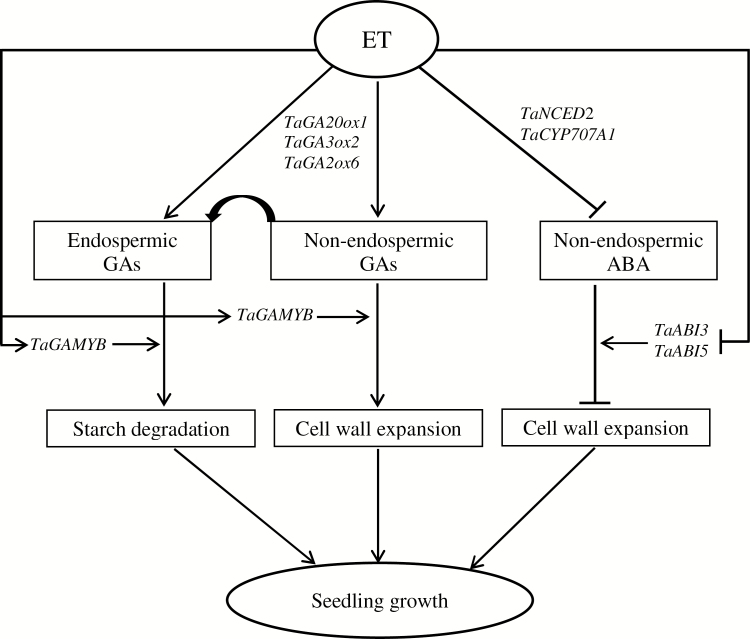
Schematic depiction of the role of ethylene (ET) in regulating abscisic acid (ABA)/gibberellin (GA) balance and seedling growth in wheat. ET enhances seedling growth by inducing endospermic and non-endospermic (embryo axis, coleoptile, and root) GA level and sensitivity through expression of GA biosynthetic (*TaGA20ox1* and *TaGA3ox2*) and/or catabolic (*TaGA2ox6*), and GA signaling (*TaGAMYB*) genes. Endospermic bioactive GA, which mainly comprises GA transported from non-endospermic tissues, induces storage starch degradation through enhancing the expression levels of specific *α-amylase* (*TaAMY1*, *TaAMY3*, and *TaAMY4*) and *α-glucosidase* (*TaAGL1*) genes and the activity of α-amylase and α-glucosidase, while GAs in non-endospermic tissues induce cell wall expansion via expression of specific *α-expansin* (*TaEXPA3*, and/or *TaEXPA7*, and/or *TaEXPA9*) genes. Furthermore, ET represses the ABA level and signaling in non-endospermic tissues via expression of ABA biosynthetic (*TaNCED*2), catabolic (*TaCYP707A1*), and ABA signaling (*TaABI3* and/or *TaABI5*) genes, contributing to the induction cell wall expansion via expression of *TaEXPA* genes. ET does not affect radicle protrusion in wheat seeds.

In summary, our study showed that ET controls storage starch degradation and seedling growth in wheat via spatiotemporal regulation of ABA and GA metabolism and signaling, and therefore the ABA/GA balance as depicted in Fig 14.

## Supplementary data

Supplementary data are available at *JXB* online.

**Fig. S1.** Effects of treatment with ethephon, gibberellin, or an abscisic acid biosynthesis inhibitor on seedling growth.

**Table S1.** Sequence similarity of starch-degrading and *GAMYB* genes with their orthologs.

**Table S2.** Gene-specific primers used for expression analysis.

**Table S3.** ABRE motifs in the promoters of *TaNCED1* and *TaNCED2* genes.

erz566_suppl_supplementary_table_S1

erz566_suppl_supplementary_table_S2

erz566_suppl_supplementary_table_S3

erz566_suppl_supplementary_figure_S1

## Abbreviations

ABA: abscisic acid
AGL: α-glucosidase
AMY: α-amylase
AVG: aminoethoxyvinylglycine
BAM: β-amylase
ET: ethylene
EXPA: α-expansin
GA: gibberellin
