# Supplementary material for: Ethylene regulates post-germination seedling growth in wheat through spatial and temporal modulation of ABA/GA balance
Source: J Exp Bot. 2019 Dec 24;71(6):1985–2004. doi: 10.1093/jxb/erz566 (PMC7094081; doi:10.1093/jxb/erz566)
Supplement: erz566_suppl_supplementary_figure_S1 [file erz566_suppl_supplementary_figure_s1.pdf]

Supplementary data – Figures

Ethylene regulates post-germination seedling growth in wheat through spatial and temporal modulation of ABA/GA balance

Menghan Sun, Pham Anh Tuan, Marta S. Izydorczyk, Belay T. Ayele

Supporting Information

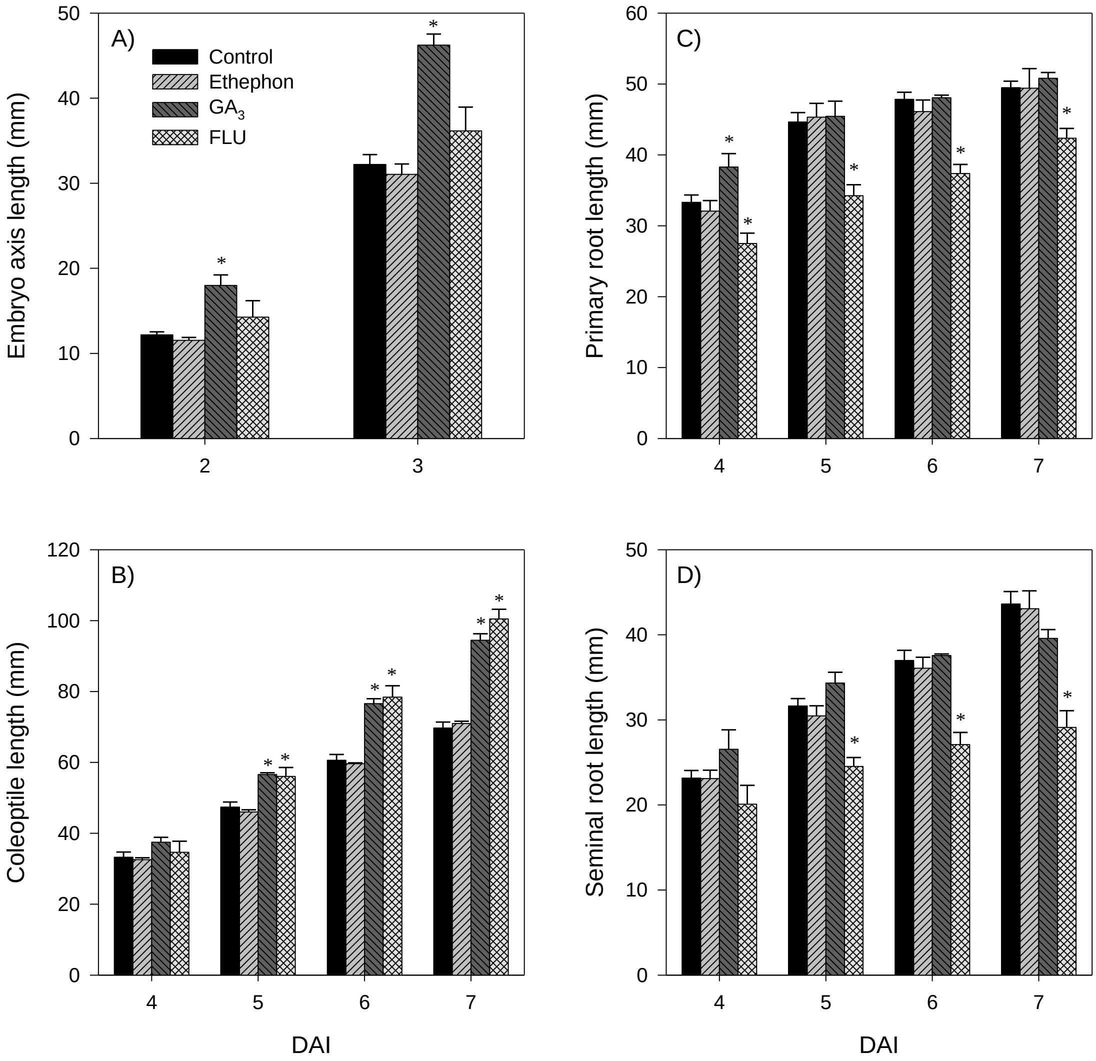

Fig. S1. Effects of treatment with ethephon or gibberellin or abscisic acid biosynthesis inhibitor on seedling growth. Lengths of embryo axis (A), coleoptile (B), primary root (C) and seminal root (D) in response to seed imbibition with ethephon (10 μM) or GA_3 (50 μM) or ABA biosynthesis inhibitor, fluridone (FLU, 50 μM). Data are means ± SE, n = 3, where n represents a batch of 20 seeds. Asterisks indicate statistically significant differences between control and treatment with ethephon or GA_3 or FLU ($P<0.05$; Student t-test). DAI, day(s) after imbibition.
